# Supplementary material for: Exploring the dimensionality of the 5C positive youth development very short form using Rasch measurement theory in Swedish upper secondary school contexts
Source: PLoS One. 2025 Dec 30;20(12):e0340051. doi: 10.1371/journal.pone.0340051 (PMC12753080; doi:10.1371/journal.pone.0340051)
Supplement: S1 Appendix — (PDF) [file pone.0340051.s001.pdf]

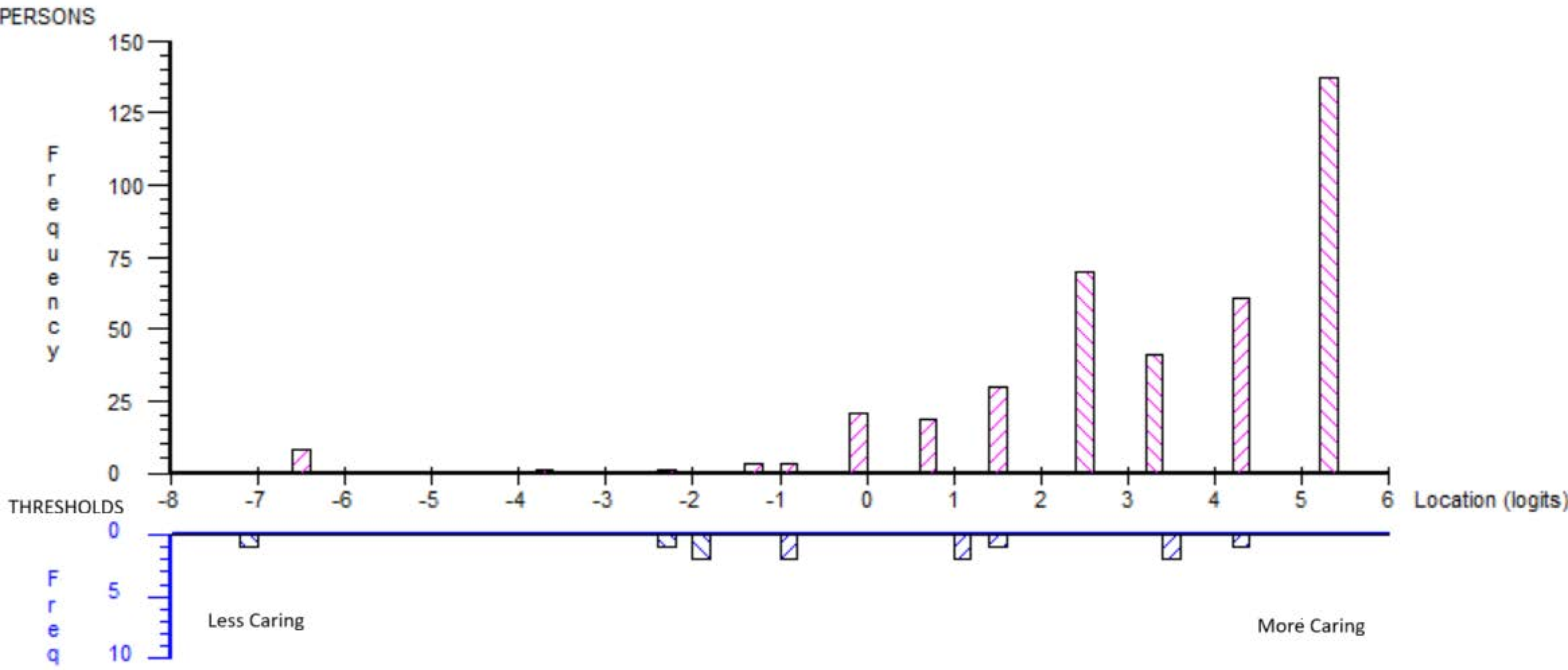
PERSONS
Frequency
150
125
100
75
50
25
0
THRESHOLDS
-8
-7
-6
-5
-4
-3
-2
-1
0
1
2
3
4
5
6
Location (logits)
Freq
0
5
10
Less Caring
More Caring

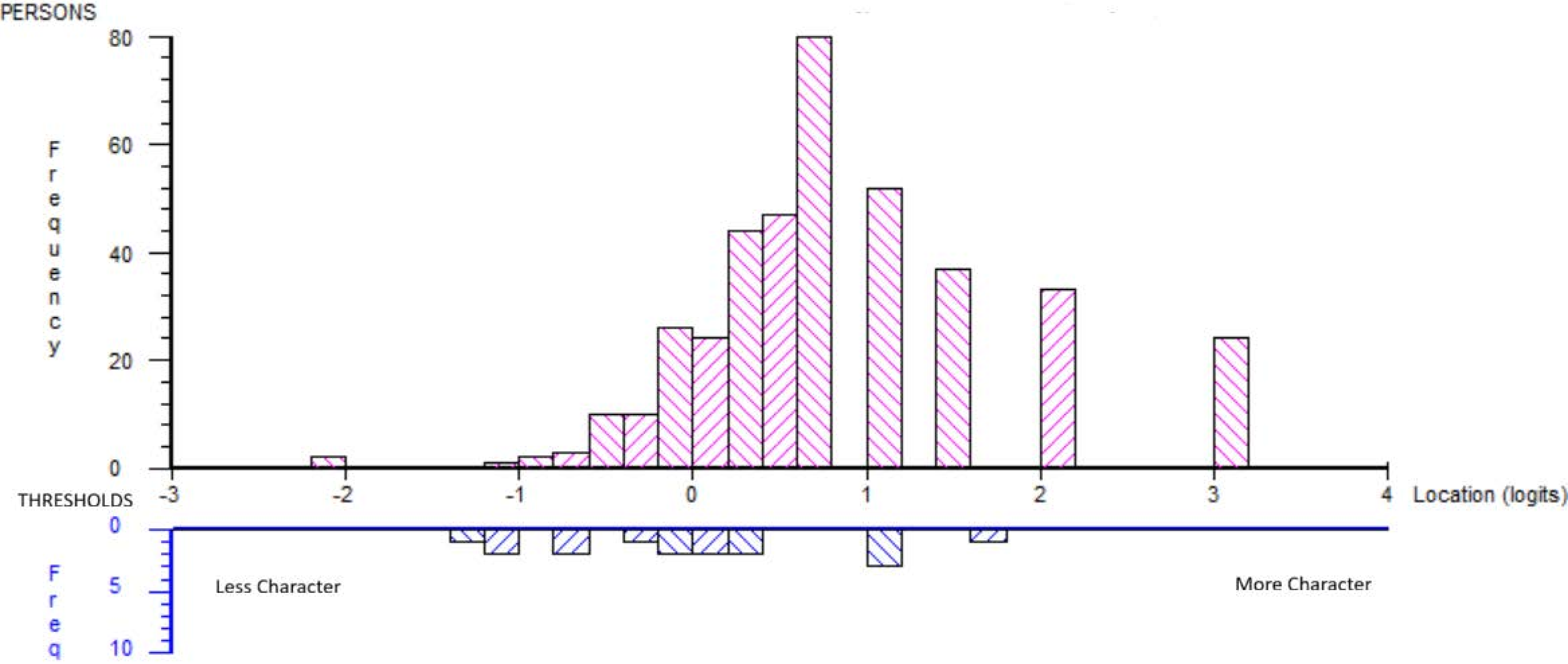
PERSONS
Frequency
80
60
40
20
0
THRESHOLDS
-3
-2
-1
0
1
2
3
4
Location (logits)
0
5
10
Freq
Less Character
More Character

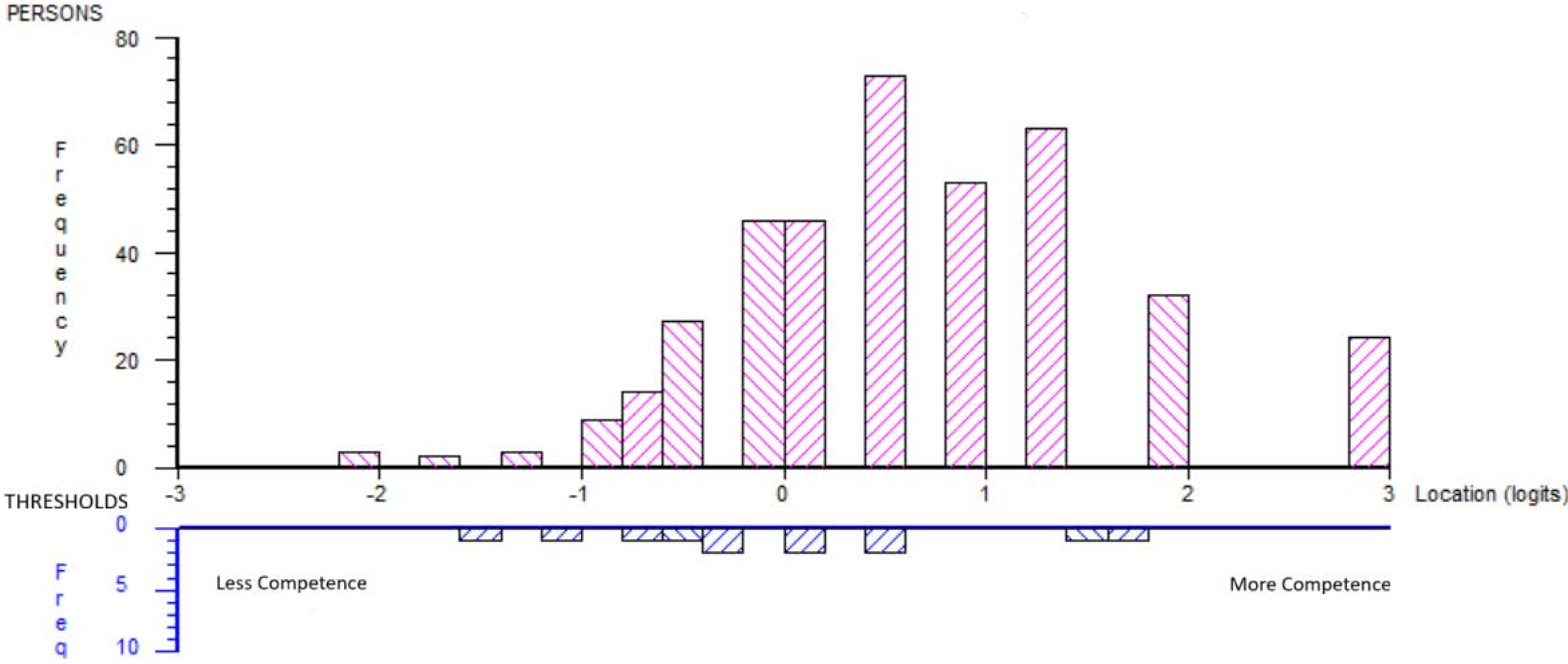
PERSONS
Frequency
80
60
40
20
0
THRESHOLDS
-3
-2
-1
0
1
2
3
Location (logits)
Freq
0
5
10
Less Competence
More Competence

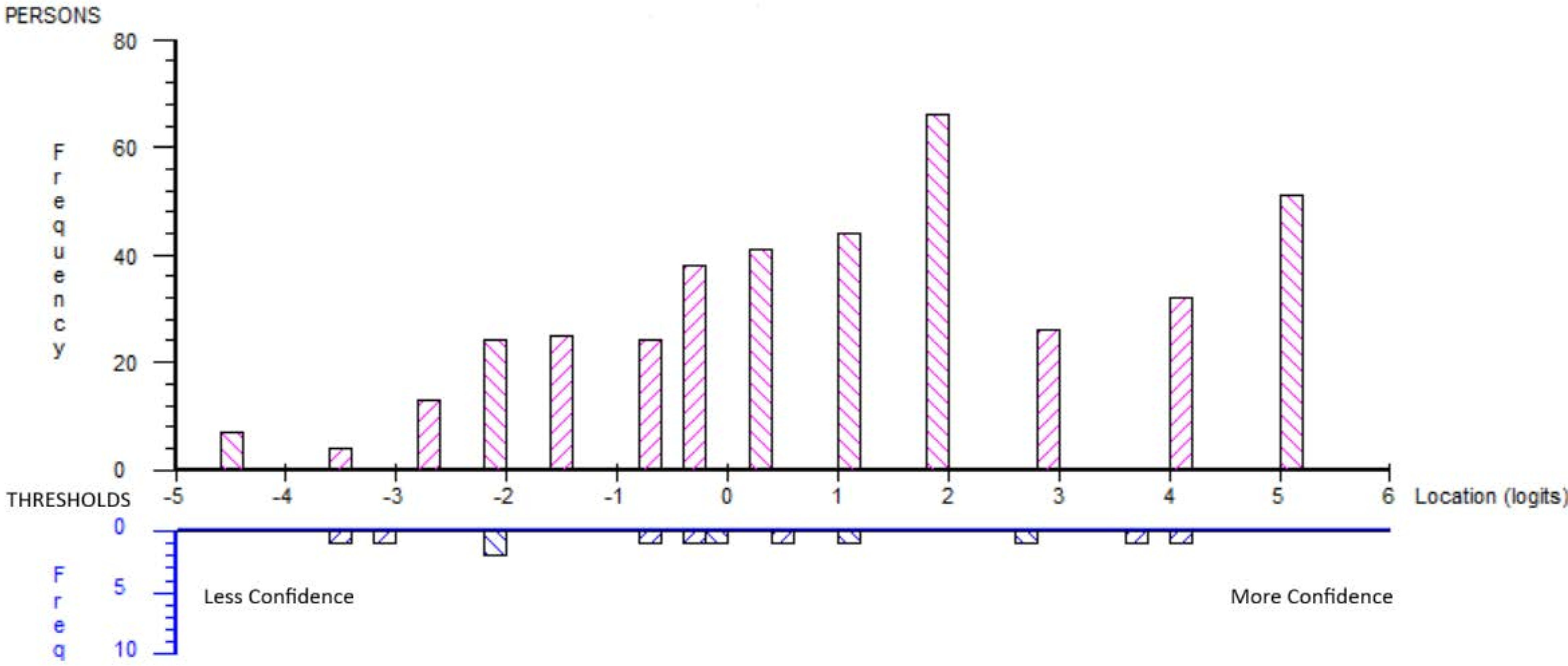
PERSONS
Frequency
80
60
40
20
0
THRESHOLDS
-5
-4
-3
-2
-1
0
1
2
3
4
5
6
Location (logits)
Freq
0
5
10
Less Confidence
More Confidence

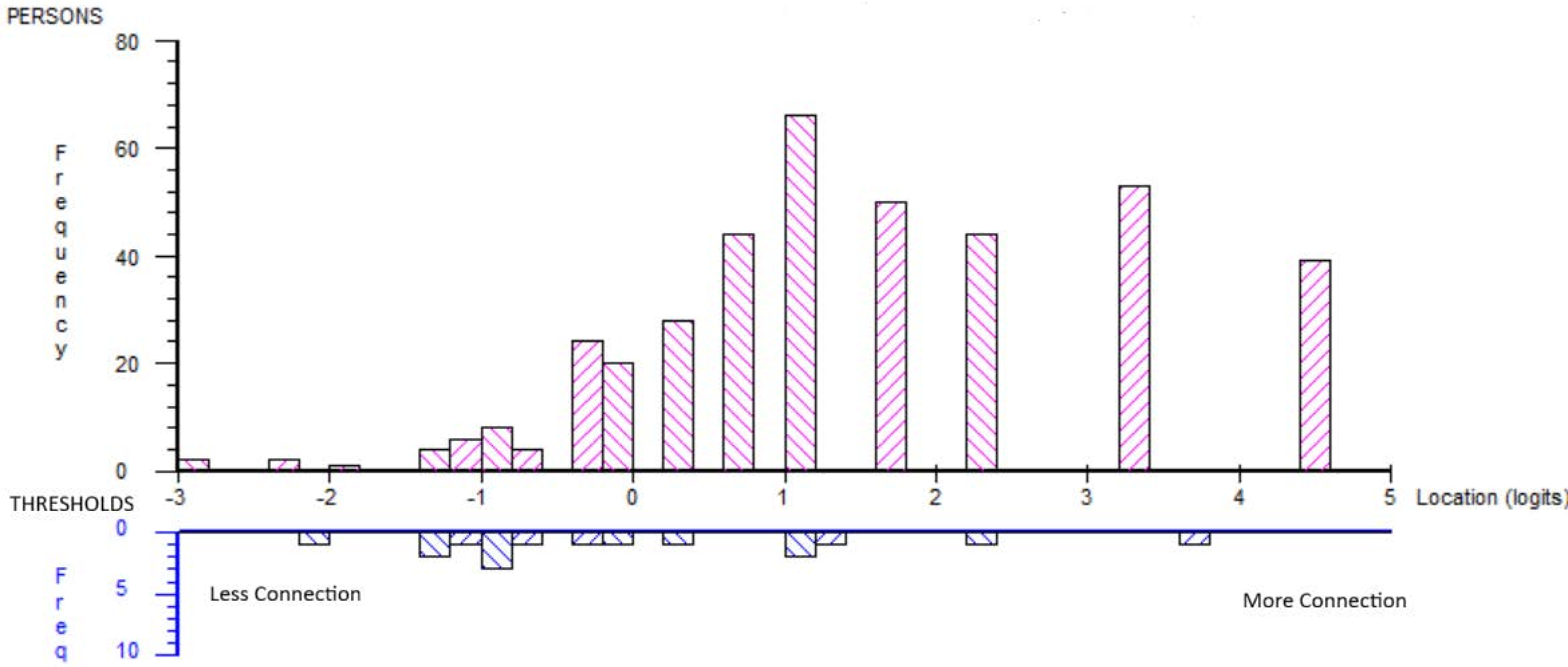

PERSONS
Frequency
80
60
40
20
0
-3
-2
-1
0
1
2
3
4
5
Location (logits)
THRESHOLDS
Freq
0
5
10
Less Connection
More Connection
