# Supplementary material for: Exploring the dimensionality of the 5C positive youth development very short form using Rasch measurement theory in Swedish upper secondary school contexts
Source: PLoS One. 2025 Dec 30;20(12):e0340051. doi: 10.1371/journal.pone.0340051 (PMC12753080; doi:10.1371/journal.pone.0340051)
Supplement: S2 Appendix — (DOCX) [file pone.0340051.s002.docx]

**Appendix S2 : Score transformation**

| APPENDIX. Translation of raw scores. Score locations to linearised scores | | | | | | | | |  |
| --- | --- | --- | --- | --- | --- | --- | --- | --- | --- |
| Self-worth | | | | | **Pro-social** | | | | |
| Raw score | Score location | Standard Error | Linearised score | Standard Error | Raw score | Score location | Standard Error | Linearised score | Standard Error |
| 0 | -3.578 | 1.09 | 0 | 5.4 | 0 | -2.66 | 0.884 | 0 | 3.8 |
| 1 | -2.926 | 0.75 | 3 | 3.7 | 1 | -2.208 | 0.656 | 2 | 2.8 |
| 2 | -2.498 | 0.583 | 5 | 2.9 | 2 | -1.888 | 0.533 | 3 | 2.3 |
| 3 | -2.217 | 0.5 | 7 | 2.5 | 3 | -1.662 | 0.465 | 4 | 2.0 |
| 4 | -2.003 | 0.448 | 8 | 2.2 | 4 | -1.479 | 0.427 | 5 | 1.8 |
| 5 | -1.828 | 0.413 | 9 | 2.0 | 5 | -1.323 | 0.399 | 6 | 1.7 |
| 6 | -1.677 | 0.387 | 9 | 1.9 | 6 | -1.183 | 0.38 | 6 | 1.6 |
| 7 | -1.541 | 0.37 | 10 | 1.8 | 7 | -1.054 | 0.367 | 7 | 1.6 |
| 8 | -1.417 | 0.355 | 11 | 1.7 | 8 | -0.931 | 0.359 | 7 | 1.5 |
| 9 | -1.302 | 0.344 | 11 | 1.7 | 9 | -0.813 | 0.353 | 8 | 1.5 |
| 10 | -1.192 | 0.335 | 12 | 1.7 | 10 | -0.696 | 0.35 | 8 | 1.5 |
| 11 | -1.086 | 0.329 | 12 | 1.6 | 11 | -0.581 | 0.35 | 9 | 1.5 |
| 12 | -0.984 | 0.325 | 13 | 1.6 | 12 | -0.464 | 0.352 | 9 | 1.5 |
| 13 | -0.883 | 0.321 | 13 | 1.6 | 13 | -0.345 | 0.355 | 10 | 1.5 |
| 14 | -0.784 | 0.319 | 14 | 1.6 | 14 | -0.221 | 0.361 | 10 | 1.6 |
| 15 | -0.686 | 0.319 | 14 | 1.6 | 15 | -0.092 | 0.368 | 11 | 1.6 |
| 16 | -0.587 | 0.319 | 15 | 1.6 | 16 | 0.044 | 0.378 | 12 | 1.6 |
| 17 | -0.488 | 0.319 | 15 | 1.6 | 17 | 0.189 | 0.389 | 12 | 1.7 |
| 18 | -0.388 | 0.321 | 16 | 1.6 | 18 | 0.343 | 0.402 | 13 | 1.7 |
| 19 | -0.286 | 0.323 | 16 | 1.6 | 19 | 0.509 | 0.417 | 14 | 1.8 |
| 20 | -0.182 | 0.326 | 17 | 1.6 | 20 | 0.688 | 0.434 | 14 | 1.9 |
| 21 | -0.076 | 0.329 | 17 | 1.6 | 21 | 0.883 | 0.456 | 15 | 2.0 |
| 22 | 0.033 | 0.333 | 18 | 1.6 | 22 | 1.099 | 0.482 | 16 | 2.1 |
| 23 | 0.144 | 0.338 | 18 | 1.7 | 23 | 1.344 | 0.516 | 17 | 2.2 |
| 24 | 0.259 | 0.343 | 19 | 1.7 | 24 | 1.627 | 0.56 | 18 | 2.4 |
| 25 | 0.378 | 0.35 | 19 | 1.7 | 25 | 1.966 | 0.621 | 20 | 2.7 |
| 26 | 0.501 | 0.357 | 20 | 1.8 | 26 | 2.396 | 0.717 | 22 | 3.1 |
| 27 | 0.629 | 0.365 | 21 | 1.8 | 27 | 3.003 | 0.899 | 24 | 3.9 |
| 28 | 0.764 | 0.374 | 21 | 1.8 | 28 | 3.861 | 1.245 | 28 | 5.3 |
| 29 | 0.906 | 0.385 | 22 | 1.9 |  |  |  |  |  |
| 30 | 1.057 | 0.398 | 23 | 2.0 |  |  |  |  |  |
| 31 | 1.22 | 0.413 | 24 | 2.0 |  |  |  |  |  |
| 32 | 1.396 | 0.431 | 25 | 2.1 |  |  |  |  |  |
| 33 | 1.589 | 0.453 | 25 | 2.2 |  |  |  |  |  |
| 34 | 1.804 | 0.48 | 27 | 2.4 |  |  |  |  |  |
| 35 | 2.047 | 0.513 | 28 | 2.5 |  |  |  |  |  |
| 36 | 2.327 | 0.556 | 29 | 2.7 |  |  |  |  |  |
| 37 | 2.662 | 0.617 | 31 | 3.0 |  |  |  |  |  |
| 38 | 3.086 | 0.713 | 33 | 3.5 |  |  |  |  |  |
| 39 | 3.687 | 0.895 | 36 | 4.4 |  |  |  |  |  |
| 40 | 4.54 | 1.245 | 40 | 6.1 |  |  |  |  |  |

Online tool for logit transformation: Ekstrand J, Westergren A, Årestedt K, Hellström A, Hagell P. (2022) Transformation of Rasch model logits for enhanced interpretability. BMC Medical Research Methodology. Doi: https://doi.org/10.1186/s12874-022-01816-1.
