# Supplementary figures and images for: Exploring the dimensionality of the 5C positive youth development very short form using Rasch measurement theory in Swedish upper secondary school contexts
Source: PLoS One. 2025 Dec 30;20(12):e0340051. doi: 10.1371/journal.pone.0340051 (PMC12753080; doi:10.1371/journal.pone.0340051)

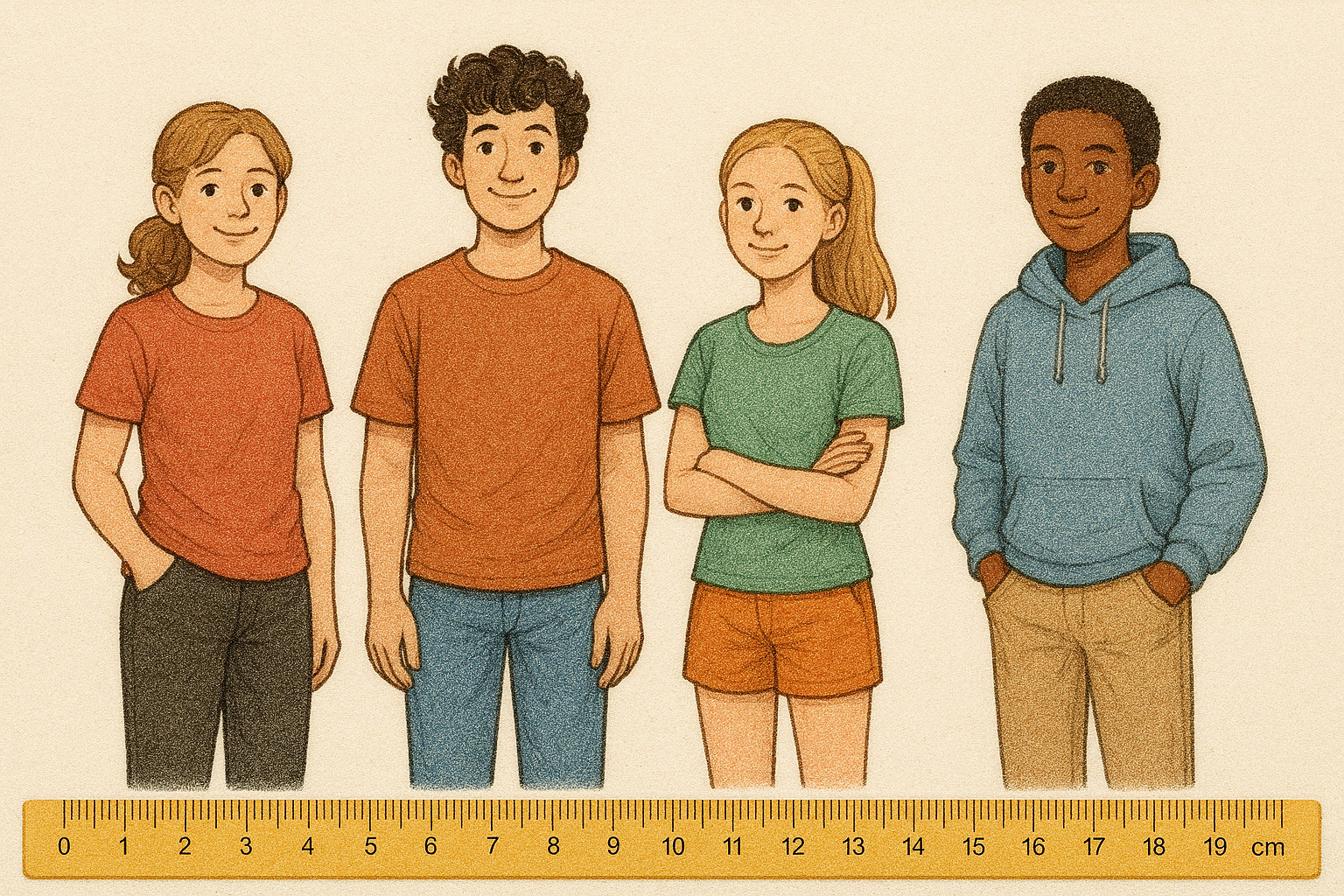

Supplement: S1 File — (TIF) [file pone.0340051.s003.tif]
